# Combined diagnosis of QF‐PCR and CNV‐Seq in fetal chromosomal abnormalities: A new perspective on prenatal diagnosis

**DOI:** 10.1002/jcla.24311

**Published:** 2022-02-23

**Authors:** Jinping Qiao, Jing Yuan, Wenjun Hu, Qin Li, Huiqin Fang, Yuanhong Xu, Yaqian Dai

**Affiliations:** ^1^ Department of Clinical Laboratory The First Affiliated Hospital of Anhui Medical University Hefei Anhui China; ^2^ Department of Obstetrics and Gynecology The First Affiliated Hospital of Anhui Medical University Hefei Anhui China

**Keywords:** CNV‐seq, karyotype analysis, prenatal diagnose, QF‐PCR, serological Down's syndrome screening

## Abstract

**Objective:**

This study aimed to evaluate the effect of QF‐PCR and CNV‐seq in diagnosing prenatal fetal chromosomal aberrations, explore the advantages and necessity of multimethod joint diagnosis.

**Methods:**

We chose pregnant women with the indication of fetal chromosome examination in our hospital last year, collected 657 cases of amniotic fluid for QF‐PCR and CNV‐seq analyzes.

**Results:**

While detecting aneuploidy, the coincidence rate of QF‐PCR and CNV‐seq was 100% (56/56). For all 46 chromosomes, 523 cases (79.60%, 523/657) coincided precisely, 128 cases (19.48%, 128/657) showed abnormality with CNV‐seq, 8 cases (1.22%, 8/657) revealed abnormality by QF‐PCR. In serological Down's syndrome screening, 328 cases showed a high risk of trisomy 21, of which CNV‐seq and QF‐PCR were consistent in 4 cases (1.22%, 4/328), CNV‐seq found 87 cases of CNVs in 78 samples except for chromosomal aneuploidy abnormalities, among these, 18 cases (20.69%, 18/87) were polymorphic, 7 cases (8.05%, 7/87) might cause disease, 13 cases (14.94%, 13/87) caused disease explicitly, 21 cases (24.14%, 21/87) were possibly benign, 17 cases (19.54%, 17/87) were explicitly benign, and the classification of 11 cases (12.64%, 11/87) was unclear.

**Conclusion:**

QF‐PCR and CNV‐seq were highly consistent in diagnosing chromosomal aneuploidy. The high risk of serological Down's screening might not only due to the aneuploidy of chromosomes 21, 18, and NTD, but also the microdeletion or microduplication of all 46 chromosomes. So using CNV‐seq combined with QF‐PCR could effectively reduce the risk of missed diagnosis.

## Graphical abstract

Copy number variation abnormalities such as microdeletion or microduplication of all 46 chromosomes might also lead to a high risk of serological Down's screening.

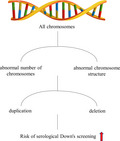

## 1 INTRODUCTION

Birth defects, also known as congenital abnormalities, are the leading cause of neonatal morbidity and death globally.^1^ Such defects refer to abnormalities in the individual, morphology, structure, and function (including metabolism, mentality, and intelligence) that occur in the uterus before the birth of the fetus but are not caused due to childbirth injuries. Such defects include congenital malformations, genetic metabolic defects, congenital disabilities (blindness, deafness, and dumbness), immune diseases, mental retardation, etc. Overall, birth defects affect approximately 1 in 33 children.^2^ According to reports, primary prevention is not possible as the cause of around 60% of birth defects is unknown, and about 20% of congenital disease are due to genetic defects.^3^ The most common genetic disease that causes birth defects in newborns is chromosomal abnormalities, accounting for about 1/160 live birth.^4^ Chromosome abnormalities, in general, include an abnormal number of chromosomes and abnormal chromosome structure. 21‐trisomy syndrome (Down syndrome), 18‐trisomy syndrome (Edward syndrome), 13‐trisomy syndrome (Patau syndrome), and sex chromosome aneuploidy (SCA) are due to a common abnormal number of chromosomes.^5^ The deletion or duplication of a tiny fragment of chromosomes is a common chromosomal structural abnormality, which is the main reason of birth defects in newborns. Nearly 300 such diseases have been found until now, such as DiGeorge, Prader–Willi, Angelman, and Williams syndrome, Williams–Beuren syndrome, 17q21.31 microdeletion syndrome, Prader–Willi and Angelman syndrome, etc.^6^, ^7^ At present, there is no effective treatment for chromosomal abnormalities. However, more and more technologies have been applied for the early diagnosis of chromosomal abnormalities, in order to achieve early prenatal intervention.

The gold standard for diagnosing fetal chromosomal abnormalities is the karyotype till now, which analyzes cells extracted from the amniotic fluid. Fetal chromosomal aneuploidy, polyploidy, abnormal balance structure, chimera, and deletions and duplications that are bigger than 10–20 Mb can be diagnosed through it. However, cell culture is required, and the method has many shortcomings such as long detection time, low throughput, and the inability to detect copy number variations (CNVs) below 5 Mb.^8^, ^9^

In recent years, there has been wide usage of genome copy number variation sequencing (CNV‐seq) technology based on low‐depth whole‐genome sequencing due to its high throughput, simple operation, and only a small sample required for the detection of chromosomal aberrations, including aneuploidy, microdeletion, microduplication, etc. Based on short tandem repeat (STR), the quantitative fluorescent polymerase chain reaction (QF‐PCR) technology has high throughput, high speed, and high accuracy and the ability to detect the contamination of maternal blood.

In this study, prenatal screening (serological Down's screening, noninvasive DNA testing, ultrasonography, etc.) and prenatal diagnosis (CNV‐seq, QF‐PCR, chromosome karyotype analysis, etc.) were carried out on 657 pregnant women with indications for chromosome examination. Then conducted a comparative analysis to explore the respective advantages of the above‐mentioned techniques and the possible clinical significance of the abnormal results.

## 2 MATERIALS AND METHODS

### 2.1 Materials

For this study, we selected pregnant women with singleton who came to our hospital for prenatal consultation from July 30, 2019 to October 23, 2020 due to abnormal serological Down's syndrome screening, high risk of noninvasive prenatal testing (NIPT), abnormal B‐ultrasound, family genetic history, adverse pregnancy history, and other factors (taking medication during pregnancy, in vitro fertilization‐embryo transform, etc.). These women signed the informed consent. We then collected 657 amniotic fluid samples and performed CNV‐seq and QF‐PCR tests at the same time. Karyotype analysis were conducted of some specimens.

### 2.2 Reagents and instruments

We purchased 21, 18, 13, and sex chromosome aneuploidy detection kits (fluorescent PCR capillary electrophoresis) from Sun Yat‐sen University Daan Gene Co., Ltd. and Guangzhou Darui Biotechnology Co., Ltd.; formamide, Liz600, ABI3500DX sequencing instrument, and corresponding Gene‐Mapper 5.0 software from Thermo Fisher Scientific Co., Ltd.; and the K5800 microspectrophotometer from Beijing Keao Company.

### 2.3 Methods

#### 2.3.1 QF⁃PCR analysis

We took 1.9 ml of amniotic fluid and centrifuged it to remove the supernatant, then used a magnetic bead method nucleic acid extraction kit (Guangzhou Darui Biotechnology Co., Ltd.) to extract the genomic DNA from the amniotic fluid and a K5800 microspectrophotometer to detect DNA quality and concentration. Then, we stored the sample at −20℃. On referring to the instructions of the corresponding kit, we detected and analyzed the samples. After amplification, we took 1 μl of the PCR product and mixed 13.5 μl formamide and 0.5 μl Liz600. Then analyzed the fragment by ABI3500DX, GeneMapper5.0, for data analysis.

#### 2.3.2 CNV⁃seq analysis

The Hunan Jiahui Genetics Specialist Hospital did CNV‐seq analysis of amniotic fluid. The experimental steps were as follows. DNA was extracted from the amniotic fluid and hydrolyzed with restriction enzymes to obtain DNA fragments with an average size of 200 bp. A library was prepared using the PCR‐free method (Beijing Beiruihe Kang Biotechnology Co., Ltd.) and connecting adapters. Then, 36 bp single‐end sequencing was done on a high‐throughput sequencing platform (NextSeq CN500 platform, Illumina), with a depth of 0.1×. All determined sequences were aligned and analyzed with the hg19 human genome through parallel alignment software (using the Burrows and Wheeler algorithm).^10^ Using 100 kb as the basic unit of analysis, the human genome was divided into several continuous regions, and the number of unique reads that matched in each region was counted. To determine the CNVs of the sample, a unique algorithm was used. According to the statistical results, the normalized sequence copy number was on the *y*‐axis. The continuous 100 kb analysis unit of each chromosome was on the *x*‐axis, drawing the CNV‐seq test result graph to determine the chromosome of the samples. The diagnostic criteria of the results were judged by the human genome hg19 version and the latest data published by DGV (http://dgv.tcag.ca/), DECIPHER (https://www.deciphergenomics.org/), OMIM (https://www.omim.org/), UCSC (https://genome.ucsc.edu/), PubMed (https://pubmed.ncbi.nlm.nih.gov/), and other databases.^11^ Duplications were defined as copy number (CN) >2.8, deletions CN <1.2, disomy (1.8 < CN < 2.2), mosaic trisomy (2.2 < CN < 2.8), and mosaic monosomy (1.2 < CN < 1.8).^12^

## 3 RESULTS

### 3.1 The classification of etiology

**FIGURE 1:**
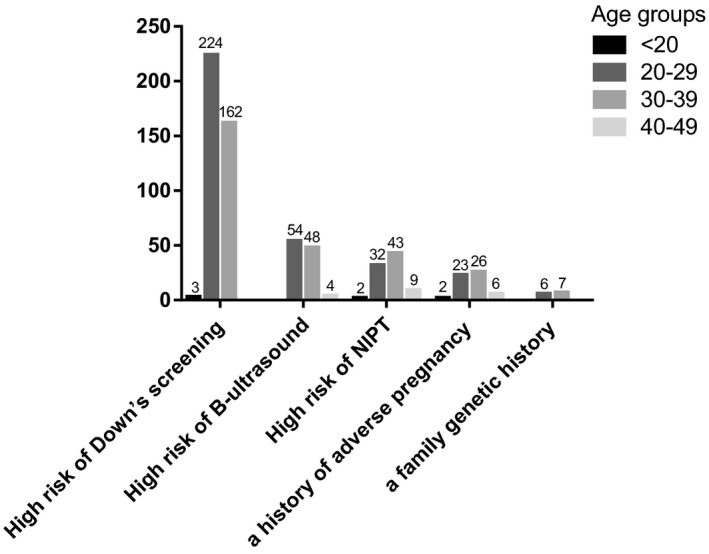
The classification of etiology

We classified the etiology of patients who underwent amniocentesis. Figure 1 showed that 389 cases (59.21%, 389/657) had a high risk of serological Down's syndrome screening: The number age below 20 years old (<20) was 3 (0.77%, 3/389); the number age between 20–29 years old (20–29) was 224 (57.58%, 224/389); the number age between 30–39 years old (30–39) was 162 (41.65%, 162/389). Abnormal B‐ultrasound accounted for 106 cases (16.13%, 106/657): 20–29 was 54 (50.94%, 54/106); 30–39 was 48 (45.28%, 48/106); 40–49 was 4 (3.77%, 4/106). We observed a high risk of noninvasive prenatal DNA testing (NIPT) in 86 cases (13.09%, 86/657): <20 was 2 (2.33%, 2/86); 20–29 was 32 (37.21%, 32/86); 30–39 was 43 (50.00%, 43/86); 40–49 was 9 (10.47%, 9/86). A history of adverse pregnancy was seen in 13 cases (1.98%, 13/657): 20–29 was 6 (46.15%, 6/13); 30–39 was 7 (53.85%, 7/13). A family genetic history accounted for 6 cases (0.91%, 6/657): 20–29 was 2 (33.33%, 2/6); 30–39 was 3 (50.00%, 3/6); 40–49 was 1 (16.67%, 1/6). Other factors (taking medication during pregnancy, in vitro fertilization‐embryo transform, etc.) were in 57 cases (8.68%, 57/657): <20 was 2 (3.51%, 2/57); 20–29 was 23 (40.35%, 23/57); 30–39 was 26 (45.61%, 26/57); 40–49 was 6 (10.53%, 6/57). Overall, the pregnancy week range was mainly concentrated below 25 weeks, among these, the pregnancy week range was below 20 weeks was 265 (40.33%, 265/657), 20–25 weeks was 246 (30.44%, 246/657), over 25 weeks was 146 (22.22%,146/657).

### 3.2 Comparison of QF‐PCR and CNV‐seq in the diagnosis of 21, 18, 13, and sex chromosome aneuploidy

CNV‐seq detected the following cases with a consistent QF‐PCR and a 100% coincidence rate: 292 cases of 46XX, 309 cases of 46XY, 3 cases of 47XX+18, 2 cases of 47XY+18, 15 cases of 47XX+21, 18 cases of 47XY+21, 1 case of 47XY+13, 4 cases of 47XXX, 7 cases of 47XXY, and 6 cases of 47XYY, as showed in Table 1. Therefore, the overall coincidence rate of QF‐PCR and CNV‐seq in the diagnosis of 21, 18, 13, and sex chromosome aneuploidy was 100%.

**TABLE 1:** Comparison of QF‐PCR and CNV‐seq in the diagnosis of 21, 18, 13, sex chromosome aneuploidy

QF‐PCR	CNV‐seq		Total
	Compatible	Incompatible	
46XX	292	0	292
46XY	309	0	309
47XX+18	3	0	3
47XY+18	2	0	2
47XX+21	15	0	15
47XY+21	18	0	18
47XY+13	1	0	1
47XXX	4	0	4
47XXY	7	0	7
47XYY	6	0	6
Total	657	0	657

Abbreviations: CNV‐seq, copy number variation sequencing; QF‐PCR, quantitative fluorescent polymerase chain reaction.

### 3.3 Comparison of QF‐PCR and CNV‐seq in the diagnosis of all chromosomal aberrations

Since QF‐PCR can only detect 21, 18, 13, sex chromosome aneuploidy, while CNV‐seq can detect all chromosomes, the coverage of QF‐PCR is far less than that of CNV‐seq. In the diagnosis of all chromosomal aberrations, 523 cases (79.60%, 523/657) showed consistent in CNV‐seq and QF‐PCR. CNV‐seq showed 128 cases (19.48%, 128/657) as abnormal, but QF‐PCR could not detect these. QF‐PCR showed 8 cases (1.22%, 8/657) as abnormal, but CNV‐seq could not detect these. The corresponding karyotypes were listed in Table 2.

**TABLE 2:** Comparison of QF‐PCR and CNV‐seq in the diagnosis of all chromosomal aberrations

Karyotype	Compatible	Abnormal by CNV‐seq	Abnormal by QF‐PCR	Total
46XX	228	60	4	292
46XY	248	59^a^	4^a^	309
46XX+21	11	4^b^	\	15
46XY+21	16	2^b^	\	18
46XX+18	3	\	\	3
46XY+18	2	\	\	2
46XY+13	1	\	\	1
46XXX	3	1^b^	\	4
46XXY	6	1^b^	\	7
46XYY	5	1^b^	\	6
Total	523	128	8	659^a^

Abbreviations: CNV‐seq, copy number variation sequencing; QF‐PCR, quantitative fluorescent polymerase chain reaction.

^a^ Two cases showed different abnormalities by CNV‐seq and QF‐PCR, so the total number was greater than the total number of cases.

^b^ Five known chromosomal aneuploidies were present, but other CNVs were positive.

### 3.4 CNVs that could not be detected by QF‐PCR but were detected by CNV‐seq

The QF‐PCR kit used in this study can only detect aneuploidies of the five chromosomes (21, 18, 13, X, and Y chromosomes). There were 141 cases CNVs existed in 128 samples that QF‐PCR could not detect but were detected by CNV‐seq, other than five chromosomal aneuploidies (among them, more than one kind of abnormal CNVs were detected in some specimens). The results are as follows: 25 cases (19.73%, 25/141) were polymorphisms, where 3 cases had CNVs located on 5 chromosomes, and 22 cases were located on other chromosomes. The disease may be caused in 15 cases (10.64%, 15/141), among which 3 cases were located on 5 chromosomes, and 12 cases were on other chromosomes. The disease could be demonstratively caused in 20 cases (14.18%, 20/141), among which 9 cases were located on 5 chromosomes, and 11 cases were on other chromosomes. There was a possibility of 32 cases (22.70%, 32/141) being benign, where 1 case was located on 5 chromosomes, and 31 cases were on other chromosomes. Twenty cases (14.18%, 20/141) were benign explicitly, where 3 cases were located on 5 chromosomes and 17 cases were located on other chromosomes. The classification of 29 cases (20.57%, 29/141) was not clear, out of which 5 cases were located on 5 chromosomes, and 24 cases were located on other chromosomes. The corresponding karyotypes were listed in Table 3.

**TABLE 3:** Pathogenicity analysis of CNVs that could not be detected by QF‐PCR but detected by CNV‐seq

Karyotypes	Polymorphisms		Pathogenic possibly		Pathogenic		Benign possibly		Benign		Unknown		Total
	5 Chromosomes	Others	5 Chromosomes	Others	5 Chromosomes	Others	5 Chromosomes	Others	5 Chromosomes	Others	5 Chromosomes	Others	
46XX	1	8	1	6	1	3	1	20	1	9	4	9	64
46XY	2	14	2	5	7	8	\	7	2	8	1	12	68
46XX+21	\	\	\	1^a^	\	\	\	1^a^	\	\	\	2^a^	4
46XY+21	\	\	\	\	\	\	\	2^a^	\	\	\	\	2
46XXX	\	\	\	\	\	\	\	\	\	\	\	1^a^	1
46XXY	\	\	\	\	1^a^	\	\	\	\	\	\	\	1
46XYY	\	\	\	\	\	\	\	1^a^	\	\	\	\	1
Total	3	22	3	12	9	11	1	31	3	17	5	24	141^b^

Abbreviations: CNV‐seq, copy number variation sequencing; QF‐PCR, quantitative fluorescent polymerase chain reaction.

^a^ Five known chromosomal aneuploidies were present, but other CNVs were positive.

^b^ One or more CNVs might be present in the same sample, so the number of CNVs was greater than the number of samples.

Among the 20 cases of pathogenic CNVs in 19 samples, 4 cases of CNVs had microdeletions or microduplications, which were >5 Mb, but the remaining 16 cases were <5 Mb, below the detection limit of karyotype analysis (5 Mb); therefore, it is impossible to detect microdeletions and microduplications smaller than 5 Mb using karyotype analysis, like the 16 cases pathogenic CNVs detected by CNV‐seq in this study. Table 4 included the results of CNV‐seq and pathogenic information.

**TABLE 4:** Pathogenic information of CNVs detected by CNV‐seq

No.	Karyotype	CNVs	Fragment size of CNVs	Disease it caused
1	46XY	seq[hg19] del (16) (p13.11) chr16:g.15140001_16280000del	1.14 Mb (microdeletions)	neurocognitive disorder susceptibility locus
2	46XX	seq[hg19] del(X) (p22.31) chrX:g.6440001_8120000del	1.68 Mb (microdeletions)	Steroid sulphatase deficiency
3	46XY	seq[hg19] del(X) (p22.31) chrX:g.6460001_8140000del	1.68 Mb (microdeletions)	Steroid sulphatase deficiency
4	46XX	seq[hg19] dup (22) (q11.21) chr22:g.18880001_21460000dup	2.58 Mb (microduplications)	22q11 duplication syndrome
5	47XX	seq[hg19] dup (7) (q21.11) chr7:g.80600001_83220000dup	2.62 Mb (microduplications)	Pontocerebellar hypoplasia, Type 3
6	46XY	seq[hg19] dup (X) (q28) chrX:g.153640001_153800000dup	0.16 Mb (microduplications)	Xq28 Microduplication syndrome
7	46XY	seq[hg19] dup (12) (p13.33p11.1) chr12:g.160001_34820000dup	34.66 Mb (microduplications)	Pallister‐Killian syndrome
8	46XY	seq[hg19] dup (3) (p14.1p13) chr3:g.68960000_70120000dup	1.16 Mb (microduplications)	Nemaline myopathy‐10
9	46XY	seq[hg19]dup(1)(q21.1q21.2) chr1:g.146500001_147760000dup	1.26 Mb (microduplications)	Recurrent microduplication
10	46XY	seq[hg19] del(Y) (p11.32q12) (mos) chrY:g.1_59373566de	59.37 Mb (microdeletions)	45, XO/46, XY Mosaic Intersex syndrome
11	46XY	seq[hg19] del(X) (p22.31) chrX:g.6460001_8080000del	1.62 Mb (microdeletions)	Steroid sulphatase (STS)
12	46XY	seq[hg19] del(X) (p22.31) chrX:g.6460000_8140000del	1.68 Mb (microdeletions)	Steroid sulphatase (STS)
13	46XY	seq[hg19] del (22) (q11.21) chr22:g.18880000_21480000del	2.60 Mb (microdeletions)	22q11 deletion syndrome
14^a^	46XX	seq[hg19] del (2) (q37.3) chr2:g.239880001_243020000del seq[hg19] dup (2) (q33.1q37.3) chr2:g.200400001_239880000dup	3.14 Mb (microdeletions) 39.48 Mb (microduplications)	2q37 monosomy syndrome Syndactyly, type 1, with or without craniosynostosis
15	46XY	seq[hg19] dup (22) (q11.21) chr22:g.18920001_21480000dup	2.56 Mb (microduplications)	22q11 duplication syndrome
16	46XY	seq[hg19] del(X) (p22.31) chrX:g.6460001_8060000del	1.60 Mb (microdeletions)	Steroid sulphatase (STS)
17	46XY	seq[hg19] del (4) (p16.3) chr4:g.40001_1800000del	1.76 Mb (microdeletions)	Growth retardation
18	46XY	seq[hg19] del(X) (p22.31) chrX:g.6460000_8140000del	1.68 Mb (microdeletions)	Steroid sulphatase (STS)
19	47XXY	seq[hg19] del(Y) (p11.2q12) chrY:g.9760001_28820000del	19.06 Mb (microdeletions)	Azoospermia Factor (Y chromosome gene)

Abbreviations: CNV‐seq, copy number variation sequencing; QF‐PCR, quantitative fluorescent polymerase chain reaction.

^a^ Two pathogenic CNVs were present in the same sample.

### 3.5 Abnormalities that could not be detected by CNV‐seq but were detected by QF‐PCR

We found 8 samples containing 9 cases of microduplications that could not be detected by CNV‐seq but were detected by QF‐PCR. The other effective STR sites on the chromosomes were all normal. However, the clinical significance was still unclear. As shown in Table 5, it needs to be diagnosed jointly with clinical and other tests.

**TABLE 5:** Abnormal information detected by QF‐PCR

No.	Karyotype	Abnormal by QF‐PCR
1	46XY	D13S634 microduplication
2	46XX	D13S634 microduplication
3	46XY	D13S634 microduplication
4	46XX	D21S1411 microduplication
5^a^	46XX	D13S628, D18S386 microduplication
6	46XX	D21S1445 microduplication
7	46XY	D13S634 microduplication
8	46XY	D13S634 microduplication

Abbreviation: QF‐PCR, quantitative fluorescent polymerase chain reaction.

^a^ Two kinds of microduplications were present in the same sample.

### 3.6 Analysis of the consistency between serological Down's syndrome screening and the combined application of CNV‐seq and QF‐PCR

As stated in Section 3.1, 389 pregnant women underwent amniocentesis in this study due to the high risk of serological Down's syndrome screening. Among them, a high risk of trisomy 21 was found in 328 cases (84.32%, 328/389), of which only 4 cases (1.22%, 4/328) were confirmed by CNV‐seq and QF‐PCR. Risk of trisomy 18 was found in 27 cases (6.94%, 27/389), but neither CNV‐seq nor QF‐PCR could detect trisomy 18. A high risk of both trisomy 21 and trisomy 18 was found in 34 cases (8.74%, 34/389); however, CNV‐seq and QF‐PCR could not detect chromosomal aneuploidy abnormalities.

When analyzing all chromosomes of 389 specimens, both CNV‐seq and QF‐PCR indicated 304 cases (78.15%, 304/389) as normal. Trisomy 21 was indicated in 4 cases (1.03%, 4/389) by CNV‐seq and QF‐PCR. QF‐PCR showed single STR site microduplication in 3 cases (0.77%, 3/389). CNV‐seq detected CNVs in 78 cases (20.05%, 78/389). Among the 3 cases that showed abnormalities through QF‐PCR, 2 cases indicated microduplication of the D13S634 site, and 1 case indicated microduplication of the D21S1411 site, among which serological Down's syndrome screening indicated a high risk of trisomy 21 simultaneously, and CNV‐seq did not prompt any abnormalities.

In Table 6, CNV‐seq found 87 cases of CNVs in 78 samples to be abnormal. Among these, 18 cases (20.69%, 18/87) were polymorphic, 7 cases (8.05%, 7/87) may cause disease, 13 cases (14.94%, 13/87) caused disease explicitly, 21 cases (24.14%, 21/87) were possibly benign, 17 cases (19.54%, 17/87) were explicitly benign, and the classification of 11 cases (12.64%, 11/87) was still unclear.

**TABLE 6:** Pathogenicity analysis of CNVs in high‐risk samples by serological Down's syndrome screening

Result	Age (years)	Polymorphisms		Pathogenic possibly		Pathogenic		Benign possibly		Benign		Unknown		Total
		5 Chromosomes	Others	5 Chromosomes	Others	5 Chromosomes	Others	5 Chromosomes	Others	5 Chromosomes	others	5 Chromosomes	Others	
High‐risk of 21 chromosome	20–25	1	3	\	\	1	\	1	1	1	3	\	1	67
	26–30	\	7	\	5	2	2	\	12	\	8	1	5	
	31–35	\	4	\	\	1	1	\	4	\	3	\	\	
High‐risk of 18 chromosome	20–25	\	\	\	\	2	\	\	1	\	\	\	\	11
	26–30	\	\	\	\	\	1	\	\	1	\	\	\	
	31–35	\	1	\	1	1	\	\	\	\	\	\	2	
High‐risk of 21 and 18 chromosomes	20–25	\	\	\	\	1	\	\	1	\	1	\	1	9
	26–30	\	2	\	\	1	\	\	1	\	\	\	\	
	31–35	\	\	1	\	\	\	\	\	\	\	\	1	
Total		1	17	1	6	9	4	1	20	2	15	1	10	87^a^

Abbreviation: CNVs, copy number variations.

^a^ One or more CNVs might be present in the same sample, so the total number was greater than the number of samples.

### 3.7 Analysis of the consistency between NIPT and the combined application of CNV‐seq and QF‐PCR

As mentioned in Section 3.1, 86 pregnant women underwent amniocentesis due to a high risk of NIPT. Out of the 86 cases, 39 cases (45.35%, 39/86) were at high risk of chromosome 21, of which 27 cases (69.23%, 27/39) were matched by CNV‐seq and QF‐PCR, 9 cases (10.47%, 9/86) were at high risk of chromosome 18, of which CNV‐seq and QF‐PCR matched 3 cases (33.33%, 3/9), 5 cases (5.81%, 5/86) were at high risk for chromosome 13, where CNV‐seq and QF‐PCR were consistent in 1 case (20.00%, 1/5), 33 cases (38.37%, 33/86) of sex chromosome were at high risk, where CNV‐seq and QF‐PCR were consistent in 13 cases (39.39%, 13/33). When it came to all chromosomes of 86 samples, 29 cases (33.72%, 29/86) were seen to be normal by CNV‐seq and QF‐PCR, 44 cases (51.62%, 37/86) indicated 5 kinds of chromosomal aneuploidy through CNV‐seq and QF‐PCR, microduplication was seen in 2 cases (2.33%, 2/86) through QF‐PCR, and CNVs were seen in 18 cases (20.93%, 18/86) through CNV‐seq. Among the 2 abnormalities suggested by QF‐PCR, 1 case was at a high risk of 18 trisomy by NIPT, and QF‐PCR indicated microduplication of D13S634 site, whereas 1 case was at a high risk of sex chromosome by NIPT and QF‐PCR indicated microduplication of D21S1445. In Table 7, we showed 20 cases CNVs in 18 samples detected by CNV‐seq. Among them, 3 cases were located on 5 chromosomes, 17 cases were located on other chromosomes. We also analyzed its pathogenicity: 2 cases (10.00%, 2/20) were polymorphic, 6 cases (30.00%, 6/20) may cause disease, 2 cases (10.00%, 2/20) caused disease explicitly, 5 cases (25.00%, 5/20) were possibly benign, and the classification of 5 cases (25.00%, 5/20) was still unclear.

**TABLE 7:** Pathogenicity analysis of CNVs in high‐risk samples by NIPT

Result	Polymorphisms		Pathogenic possibly		Pathogenic		Benign possibly		Benign		Unknown		Total
	5 Chromosomes	Others	5 Chromosomes	Others	5 Chromosomes	Others	5 Chromosomes	Others	5 Chromosomes	Others	5 Chromosomes	Others	
High‐risk of 21 chromosome	\	1	\	1		2	\	3	\	\	\	4	11
High‐risk of 18 chromosome	\	\	2	1	\	\	\	\		\	\	\	3
High‐risk of 13 chromosome	\	\	\	1	\	\	\	\	\	\	\	\	1
High‐risk of sex chromosomes	1	\	\	1	\	\	\	2	\	\	\	1	5
Total	1	1	2	4	0	2	0	5	0	0	0	5	20^a^

Abbreviations: CNVs, copy number variations; NIPT, noninvasive prenatal testing.

^a^ One or more CNVs might be present in the same sample, so the total number was greater than the number of samples.

## 4 DISCUSSION

Each year, there have been roughly 135 million newborns worldwide, of which 3% suffer from major structural birth defects. This brings a serious economic and spiritual burden to society and the family.^13^ Therefore, diagnosing fetal chromosomal aberrations quickly and accurately has become more and more important to eliminate the mother's anxiety and reduce the birth rate of abnormal fetuses.

Till now, karyotype analysis is the gold standard for prenatal diagnosis of fetal chromosomal aberrations. However, there are many shortcomings, such as long detection time, cell culture requirement, low resolution (<5 Mb), and misdiagnosis due to maternal blood contamination.^14^ CNV‐seq technology has been widely used in detecting chromosomal aberrations such as aneuploidy, microdeletion, and microduplication due to its many advantages, such as high throughput and easy operation. However, it cannot identify maternal cell contamination. QF‐PCR can diagnose common chromosomal aneuploidy within 24 h, through qualitative and quantitative analysis of the polymorphism of STR genetic markers, adopt multiplex PCR amplification and capillary electrophoresis separation technology. One of the advantages of QF‐PCR is that it can identify maternal contamination. However, it cannot detect chromosome structure abnormalities, chromosome polyploidy, and mosaics with a mosaic ratio that is <20%.^15^

Among the 657 amniotic fluid specimens in this study, while diagnosing aneuploidy in 5 chromosomes (13, 18, 21, X, and Y), we found 33 cases of trisomy 21, 5 cases of trisomy 18, 1 case of trisomy 13, 4 cases of XXX, 7 cases of XXY, and 6 cases of XYY. The coincidence rate between CNV‐seq and QF‐PCR was 100%. For all chromosomal structure and number abnormalities, the rate of QF‐PCR and CNV‐seq was the same at 79.60%. The rate of abnormality indicated by CNV‐seq but not QF‐PCR was 19.48%, whereas that indicated by QF‐PCR but not CNV‐seq was 1.22%.

Among the 9 cases abnormalities that could not be detected by CNV‐seq but QF‐PCR, 5 cases showed D13S634 site microduplication, accounting for 55.56%. In the remaining, D21S1411, D13S628, D18S386, and D21S1445 site microduplication were seen in 1 case each, accounting for 11.1%, respectively. Although the clinical significance was still not clear, it still showed guiding significance for clinical auxiliary diagnosis and future prenatal chromosomal aberration detection.

Among the 141 CNVs that could not be detected by QF‐PCR but CNV‐seq, 17.73% were polymorphic, the pathogenicity of 20.57% was unknown, 22.7% were possibly benign, 14.18% were explicitly benign, 10.64% were possibly pathogenic, and 14.18% were pathogenic. Regarding the size of chromosomal CNVs variant fragments, 4.26% were larger than 5 Mb, and 95.74% were <5 Mb, which was less than the resolution of the karyotype. If only the karyotype analysis was performed, it might lead to a missed diagnosis.

There were 389 cases (59.21%, 389/657) in this study who underwent amniocentesis due to a high risk of serological Down's syndrome screening, which accounted for the largest proportion. Serological Down's syndrome screening determines the fetus's risk factor that may suffer from 21‐trisomy syndrome (Down Syndrome), 18‐trisomy syndrome (Edward Syndrome) and neural tube defects (NTD). In combination with the age, weight, and gestational age of the pregnant woman, it analyzes the concentration of fetal alpha‐fetal protein, chorionic gonadotropin, and free estriol in the maternal serum.^16^ CNV‐seq and QF‐PCR only detected 4 cases of trisomy 21 among the 328 cases, which were at high risk of trisomy 21 by serological Down's syndrome screening, with a coincidence rate of 1.22% (4/328). Among the population with a high risk of trisomy 18, no positive case was detected, and the accuracy was relatively low.

However, among the 389 cases, which had a high risk of serological Down's screening, there were 78 cases (20.05%, 78/389) detected abnormal CNVs but without chromosomal aneuploidy abnormalities by CNV‐seq. Therefore, when these pregnant women were aware of the high risk of serological Down's screening, if they only chosen NIPT to further verify whether there were 21‐trisomy syndrome or 18‐trisomy syndrome but gave up amniocentesis for prenatal diagnosis based on amniotic fluid, the result of NIPT should be that the fetus were normal, the existing CNVs would be missed.

However, judging from the abnormality of CNVs, out of 389 fetuses with a high risk of serological Down's screening (high risk of trisomy 21 and trisomy 18), only 4 cases (1.03%, 4/389) were diagnosed as trisomy 21. But there were 78 cases (20.05%, 78/389) existing 87 kinds of CNVs, which were characterized by microdeletion and microduplication (there might be two or more CNVs in the same specimen, so the number of CNVs was greater than the total number of samples), of which 13 cases were pathogenic, 39 cases were pathogenic possibly, benign possibly or the significance was unclear temporarily, 35 cases were known to be benign and polymorphic. And among these 13 cases CNVs, which were pathogenic, the fragment size of 2 cases were larger than 5 Mb, and 11 cases were <5 Mb, suggesting that if these 11 cases were diagnosed by karyotype analysis based on amniotic fluid, might cause missed diagnosis because of the detection limit (<5 Mb). This ratio was much higher than our understanding of the positive rate of serological Down's screening. Therefore, this result suggests that the high risk of serological Down's screening may not only due to the aneuploidy of chromosomes 21 and 18, but also the microdeletion or microduplication of all chromosomes (including chromosomes 21 and 18). In addition, we need to remind everyone that it might cause missed diagnosis if only NIPT was used for further detection for people who were at the high risk. Through amniocentesis, using CNV‐seq and other high‐resolution, high‐coverage prenatal diagnosis technology for prenatal diagnosis, can detect the abnormalities of these CNVs that may cause disease to the greatest extent.

86 (13.09%, 86/657) cases underwent amniocentesis because a high risk of NIPT. It sequences free DNA fragments (including free fetal DNA) in maternal peripheral plasma using next‐generation DNA sequencing technology and then analyzes the result by biologic information.^17^ Among the 86 cases in this study, the coincidence rate of chromosome 21 was 69.23% (27/39), the coincidence rate of chromosome 18 was 33.33% (3/9), the coincidence rate of chromosome 13 was 20.00% (1/5), and the coincidence rate of sex chromosomes was 39.39% (13/33). The accuracy rate was improved compared to serological Down's syndrome screening, but it is still a screening experiment that could not be made for a final diagnosis. In addition, the accuracy of NIPT decreased correspondingly in the following situations: if the pregnancy week was too early or too late, if the expected age was ≥35 years, if the pregnant woman was severely obese (body mass index >40), etc.^18^

In conclusion, QF‐PCR can detect the contamination of maternal cells, it possesses high sensitivity and specificity in diagnosing aneuploidy in 5 chromosomes of 13, 18, 21, X, and Y within a short time, but it cannot detect abnormal chromosomal structure and low‐proportion mosaicism. For these reasons, CNV‐seq can be better supplemented, it can detect chromosomal aberrations such as microdeletion and microduplication, which were <5 Mb. However, QF‐PCR is still required for distinguishing whether existing maternal cell contamination. In addition, it should be noted that CNVs such as microdeletion or microduplication of all chromosomes (including chromosomes 21 and 18) may cause a high risk of serological Down's screening except the existence aneuploidy of chromosomes 21, 18 and NTD, which may cause missed diagnosis if only NIPT was used for further detection. CNV‐seq combined with QF‐PCR can complement each other in order to diagnose fetal chromosomal abnormalities more efficiently and accurately, this combination may be considered as the first‐line method of prenatal chromosome diagnosis.

### 4.1 Limitation statement about the research

This research based on a small sample size, so the conclusion might have limited generalizability, we will continue to collect samples in future to get more convincing results. In addition, our research is only for Chinese people, and we encourage scientists from other countries to also participate in this research.

## AUTHOR CONTRIBUTIONS

All authors have accepted responsibility for the entire content of this manuscript and approved its submission.

## COMPETING INTERESTS

Authors state no conflict of interest.

## INFORMED CONSENT

Not applicable.

## Data Availability

The data that support the findings of this study are available from the corresponding author (dyq13655602140@163.com) upon reasonable request.
